# Oropouche fever in Minas Gerais, Brazil, 2024: Epidemiological characterization, spatial distribution, and clinical implications

**DOI:** 10.1590/0037-8682-0109-2025

**Published:** 2026-03-02

**Authors:** Edmundo Rinolino Magalhães Flores, Flavia Ribeiro Soares Cruzeiro, Iara Fabíola Rocha, Caroline Macedo Gonçalves, Thais Cristina Nazaré Freitas, Daniela Caldas Teixeira

**Affiliations:** 1 Secretaria de Estado de Saúde de Minas Gerais, Centro de Informações Estratégicas em Vigilância em Saúde, Belo Horizonte, MG, Brasil.; 2 Universidade Federal de Minas Gerais, Programa de Pós-Graduação Stricto Sensu em Neurociências, Belo Horizonte, MG, Brasil.; 3 Universidade Federal de Minas Gerais, Departamento de Pediatria, Belo Horizonte, MG, Brasil.

**Keywords:** virus, Arbovirus, Epidemiology, Public health surveillance, Spatial analysis

## Abstract

**Background:**

Oropouche fever (OF), caused by the Oropouche virus (OROV), has historically been endemic to the Amazon region; however, it is now emerging in other Brazilian states. This study describes the sociodemographic, clinical, and spatial characteristics of OROV cases identified in Minas Gerais in 2024 and contributes to the understanding of its transmission dynamics in non-endemic regions.

**Methods:**

This descriptive epidemiological study used the official surveillance data from the State Health Department of Minas Gerais (SES/MG). The confirmed cases were those with laboratory-confirmed OROV infection by RT-PCR at the Ezequiel Dias Foundation (FUNED). Demographic, geographic, and clinical data were analyzed using descriptive statistics. Exploratory comparisons by sex and age were performed.

**Results:**

A total of 278 confirmed autochthonous cases were reported across 10 municipalities, primarily in rural and peri-urban areas. Women accounted for 55.0% of the cases, with the highest incidence among adults aged 20-59 years. Two temporal peaks were observed at epidemiological weeks 15 and 50, corresponding to distinct clusters in Joanésia and Piau, respectively. Symptom recurrence occurred in 25.5% of the cases. Headache, myalgia, gastrointestinal symptoms, dysgeusia, pruritus, and skin lesions were more common in women than in men. Hypertension and diabetes mellitus were the most common comorbidities. More than half the participants reported the presence of domestic animals nearby.

**Conclusions:**

This study confirmed the geographic expansion of OROV in southeastern Brazil and identified distinct temporal and spatial transmission patterns. These findings underscore the importance of strengthened arbovirus surveillance, improved laboratory capacity, and integrated vector control strategies.

## INTRODUCTION

Oropouche fever (OF) is an acute febrile illness caused by the Oropouche virus (OROV), an arbovirus belonging to the*Orthobunyavirus*genus of the*Peribunyaviridae*family^1^ ^,^ ^2^ ^.^ The virus is primarily transmitted through the bite of*Culicoides paraensis*and circulates in two transmission cycles, urban and sylvatic, which may also involve*Aedes*and*Culex*mosquitoes as well as vertebrate hosts such as primates and birds^3^ ^,^ ^4^. Since its identification in the 1960s, more than 500,000 human cases have been estimated to have occurred, although the true burden is possibly higher because of underdiagnosis and the clinical similarity of OF to other arboviral infections, such as dengue, Zika, and chikungunya^5^ ^,^ ^6^.

Oropouche fever is endemic to the humid tropical regions of Central and South America, including Brazil, and has historically shown a higher incidence in the Amazon region. However, reports from other states such as Minas Gerais and São Paulo indicate a geographic expansion of OROV transmission^7^ ^,^ ^8^. Although the disease is generally self-limiting, it can result in prolonged or recurrent symptoms, impaired quality of life, and productivity losses^9^ ^,^ ^10^. In addition, outbreaks in resource-limited settings pose challenges for local health systems.¹ In addition, limited access to specific diagnostic tools and gaps in clinical and epidemiological data hinder accurate case detection and the development of effective control strategies^11^ ^,^ ^12^.

According to the 2024 epidemiological update for the Region of the Americas^13^, 16,239 confirmed OF cases have been reported, including four deaths. The disease has occurred in 11 countries, including the Cayman Islands, with Brazil accounting for 13,785 cases and all reported deaths. The Amazon region, where OROV is endemic, accounts for 42.0% of Brazilian cases. Nonetheless, detection of the virus in regions outside the Amazon has led to confirmed infections in more than 15 states, including Minas Gerais. Fatal cases were reported in Bahia, Paraná, and Espírito Santo.

Given the recent detection of OROV in non-endemic areas, this study aims to describe the epidemiological profile of confirmed OF cases in Minas Gerais by 2024, thereby contributing to improved surveillance, case management, and public health policy planning in Brazil. In addition, exploratory analyses were performed to compare the frequency of key symptoms and comorbidities according to sex and age, with the aim of providing a more detailed clinical characterization of confirmed cases.

## METHODS

### Study design

This descriptive study analyzed all confirmed cases of OROV infection in Minas Gerais, Brazil. The data presented in this study correspond to cases with symptom onset dates beginning in 2024. Data were extracted on February 10, 2025, using the official health surveillance information system. Subsequent updates to the database may result in changes to the reported numbers. Therefore, comparisons with data extracted at different time points should account for potential modifications due to the ongoing data validation and consolidation processes.

In 2024, OF cases were reported exclusively upon laboratory confirmation by molecular biology (RT-PCR), as no official definition of a suspected case had yet been established. Confirmed cases were reported via GoData and registered in the*Sistema de Informação de Agravos de Notificação*(SINAN).

This observational study followed the recommendations of the Strengthening the Reporting of Observational Studies in Epidemiology (STROBE) statement^14^.

Minas Gerais comprises 853 municipalities distributed over a territorial area of 586,513.984 km² and has an estimated population of 20,539,989 inhabitants as of 2023, making it the second most populous state in Brazil. For administrative and epidemiological purposes, the State Health Department of Minas Gerais (SES/MG) divided the territory into 28 Regional Health Units (RHU) as established by SES/MG Resolution No. 9,224 on December 12, 2023.

### Sample and surveillance procedures

Following the detection of OF cases outside the Amazon region in May 2024, the Ezequiel Dias Foundation (FUNED), in collaboration with SES/MG, developed a sampling and laboratory testing protocol to identify potential cases in Minas Gerais. In this study, confirmed cases were strictly defined as individuals with laboratory-confirmed OROV infection by RT-PCR. The inclusion criteria were as follows: (i) residence in Minas Gerais, (ii) availability of complete sociodemographic and clinical data from GoData, and (iii) laboratory confirmation at FUNED. The exclusion criteria included incomplete records or indeterminate RT-PCR results.

Sentinel surveillance of arboviruses will be implemented in June 2024. Based on the initial detection of OROV-positive samples by RT-PCR, the diagnostic investigation was expanded beyond the sampling strategy to include broader local surveillance in the municipalities with identified cases.

Once cases were confirmed, standardized OROV investigation forms were completed in GoData by municipal health teams following the same procedures routinely adopted for other notifiable diseases in Brazil. All information analyzed in this study originated from official surveillance records, which were filled out by local health professionals during the case investigation as part of their public health duties. The authors did not conduct interviews or collect primary data. Rather, they accessed de-identified secondary data extracted from the GoData system, which includes more detailed fields than the standard SINAN notification forms.

### Variables

The sociodemographic variables analyzed included sex, age, race/skin color, educational level, municipality of residence, RHU, and area of residence (urban or rural). Clinical variables included the presence of signs and symptoms, symptom duration, date and epidemiological week of symptom onset, symptom recurrence, and the presence of comorbidities. Epidemiological context variables included the presence of animals in the peridomicile, reports on epizootics, and other relevant environmental observations.

### Data analysis

Data were exported from GoData in the CSV format and subsequently analyzed using IBM® SPSS 21.0.0.0. Numerical variables are summarized using measures of central tendency (mean or median) and dispersion (standard deviation or interquartile range), according to their distribution. Categorical variables are described as absolute and relative frequencies. Graphs and tables were used to visualize the temporal and spatial trends in OROV cases across Minas Gerais. The incidence rates per 100,000 inhabitants were calculated using the estimated population for Minas Gerais in 2024, published by the Instituto Brasileiro de Geografia e Estatística (IBGE) in Diário Oficial da União on August 29, 2024.

In addition to descriptive statistics, exploratory analyses were conducted to compare the distribution of selected symptoms by sex and age. These symptoms were selected because of their high prevalence and clinical relevance. A chi-square test was used to explore the relationship between the presence of symptoms and sex. Associations between sex and symptoms were estimated using Poisson regression with robust variance to obtain prevalence ratios (PRs) and 95% confidence intervals. For age comparisons, the Mann-Whitney *U* test was applied (because the data did not present a normal distribution). One patient was excluded from the analysis because of missing information. The Chi-square test was used to assess differences in proportions between groups, as is commonly recommended for categorical data. In addition, PR with 95% confidence intervals was estimated using Poisson regression with robust variance to quantify the magnitude of the associations. All analyses were performed using IBM SPSS Statistics, version 21.0.0.0.

### Ethical considerations

This study was approved by the Research Ethics Committee of the Hospital Foundation of the State of Minas Gerais (approval number: 7,399,279). Because it relied exclusively on secondary de-identified data from official surveillance systems, it complied with the principles outlined in Resolution No. 466/2012 of the Brazilian National Health Council, ensuring the confidentiality, privacy, and ethical use of epidemiological information.

## RESULTS

**FIGURE 1:**
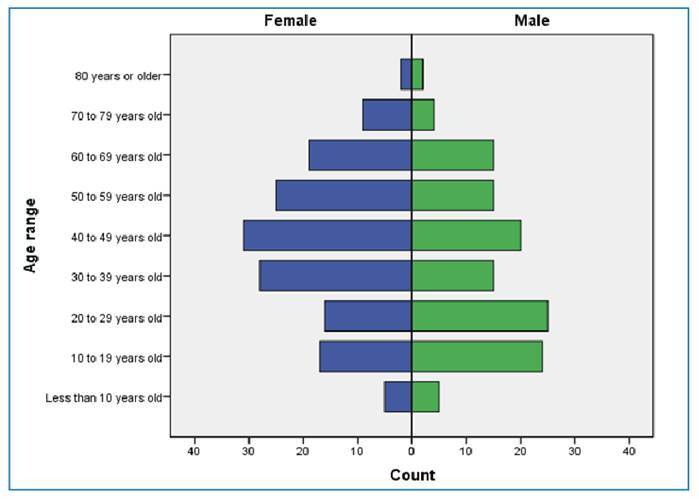
Confirmed Oropouche fever cases by sex and age group, Minas Gerais, Brazil, 2024 (*N* = 278). **Note:** This figure presents the data on cases reported in 2024, extracted from the official surveillance system on February 10, 2025.

In 2024, 278 confirmed cases of OF were reported in Minas Gerais. Most cases were observed in females (55.0%). The mean age of the affected individuals was 40.14 years (SD ± 19.19), with a median age of 40 years (interquartile range [IQR]: 25-56) (Table 1; Figure 1). Most patients self-identified as mixed race (46.8%) or White (32.4%), and over half (54.7%) had completed elementary education.

**TABLE 1:** Sociodemographic profile of confirmed Oropouche fever cases, Minas Gerais, Brazil, 2024 (N = 278).

Characteristics	Confirmed cases (%)
**Sex**	
Female	153 (55.0)
Male	125 (45.0)
**Race/Color**	
Asian	4 (1.4)
White	90 (32.4)
Mixed-race	130 (46.8)
Black	38 (13.7)
**Education level**	
No schooling/Incomplete elementary school	103 (37.0)
Complete elementary school/Incomplete high school	49 (17.6)
Complete high school/Incomplete vocational or higher education	86 (30.9)
Complete technical/vocational or higher education	20 (7.2)
**Regional health unit/Municipality of residence**	
**Barbacena**	
Congonhas	1 (0.4)
**Coronel Fabriciano**	
Coronel Fabriciano	30 (10.8)
Ipatinga	3 (1.1)
Joanésia	140 (50.4)
Timóteo	15 (5.4)
**Governador Valadares**	
Coroaci	1 (0.4)
Gonzaga	1 (0.4)
**Juiz de Fora**	
Bom Jardim de Minas	1 (0.4)
Piau	84 (30.2)
Ubá	
Tabuleiro	2 (0.7)
**Residential area**	
Peri-urban	78 (28.1)
Rural or sylvatic	106 (38.1)
Urban	74 (26.6)

Note: The number of cases in certain categories may vary due to missing information. This table presents the data on cases reported in 2024, extracted from the official surveillance system on February 10, 2025.

Two cases of OF were diagnosed in pregnant women, both in the second trimester and without comorbidities. One resided in Joanésia and the other in Piau. The reported symptoms included fever, headache, myalgia, arthralgia, chills, nausea, vomiting, dysgeusia, ageusia, photophobia, dizziness, pruritus, fatigue, malaise, and ecchymosis. Both patients received regular prenatal care, and no maternal or fetal complications were reported.

The most frequent symptoms among confirmed cases were fever, headache, myalgia/arthralgia, gastrointestinal manifestations, and retroorbital pain (Table 2). Upper respiratory tract symptoms such as nasal discharge, odynophagia, and cough were less common. Dermatological findings included petechiae, ecchymosis, rash, and erythema, whereas gastrointestinal symptoms included nausea, diarrhea, vomiting, and abdominal pain.

**TABLE 2:** Clinical profile of confirmed Oropouche fever cases, Minas Gerais, Brazil, 2024 (*N* = 278).

Characteristics	Cases (%)
**Signs and symptoms**	
Fever	231 (83.1)
Headache	212 (76.3)
Myalgia	196 (70.5)
Gastrointestinal symptoms	164 (59.0)
Retro-orbital pain	140 (50.4)
Ageusia	137 (49.3)
Arthralgia	128 (46.0)
Chills	120 (43.2)
Dysgeusia	120 (43.2)
Dizziness	92 (33.1)
Fatigue or weakness	87 (31.3)
Malaise	79 (28.4)
Back pain	77 (27.7)
Pruritus	76 (27.3)
Skin lesions	58 (20.9)
Photophobia	30 (10.8)
Flu-like or upper respiratory symptoms	28 (10.1)
Nuchal pain	28 (10.1)
Conjunctivitis	15 (5.4)
Dark urine	14 (5.0)
Shortness of breath	13 (4.7)
Oliguria	7 (2.5)
Jaundice	5 (1.8)
Sensitivity to touch	5 (1.8)
Leg pain	5 (1.8)
Tachycardia	2 (0.7)
**Comorbidities**	
Hypertension	65 (23.4)
Diabetes	28 (10.1)
Heart disease	6 (2.2)
Lung disease (chronic obstructive pulmonary disease, asthma, tuberculosis)	5 (1.8)
Hematological diseases	3 (1.1)
Chronic kidney disease	2 (0.7)

**Note:** This table presents the data on cases reported in 2024 extracted from the official surveillance system on February 10, 2025.

**FIGURE 2:**
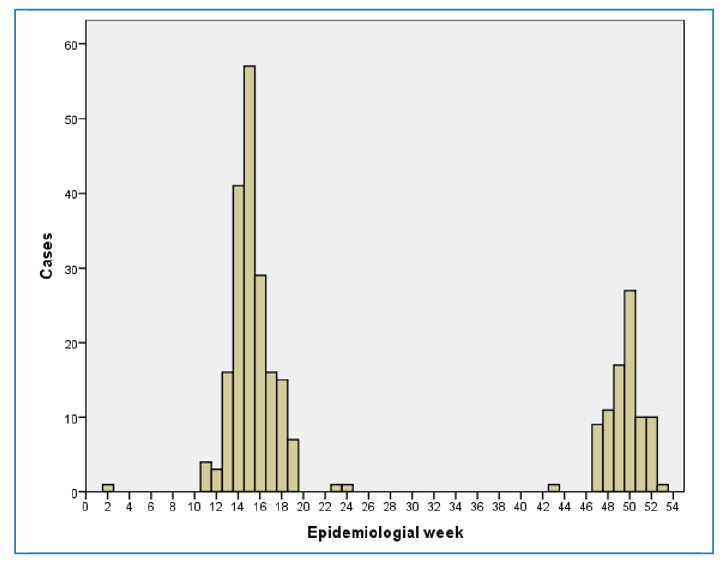
Confirmed OF cases by epidemiological week of symptom onset, Minas Gerais, Brazil, 2024 (*N* = 278). **Note:** This figure presents the data on cases reported in 2024, extracted from the official surveillance system on February 10, 2025.

The temporal distribution of cases demonstrates two distinct peaks in epidemiological weeks 15 and 50 in 2024 (Figure 2). These peaks coincided with the detection of outbreaks in Joanésia and Piau, municipalities with the largest number of confirmed cases during the study period.

The mean symptom duration was 6.77 days (SD ± 5.25), with a median of 5 days (IQR, 4-7). Symptom recurrence was reported in 71 cases (25.5%), with an average time of reappearance of 9.88 days (SD ± 13.57) and a median of 5 days (IQR: 3-10).

The most frequent comorbidities among the confirmed cases were hypertension (23.4%) and diabetes mellitus (10.1%) (Table 2). Other comorbidities were less common and included chronic respiratory diseases (COPD, asthma, or tuberculosis), cardiovascular conditions, hematologic disorders, chronic kidney disease, leprosy, dyslipidemia, gastritis, depression, anxiety, migraines, hypothyroidism, and musculoskeletal disorders.

**FIGURE 3:**
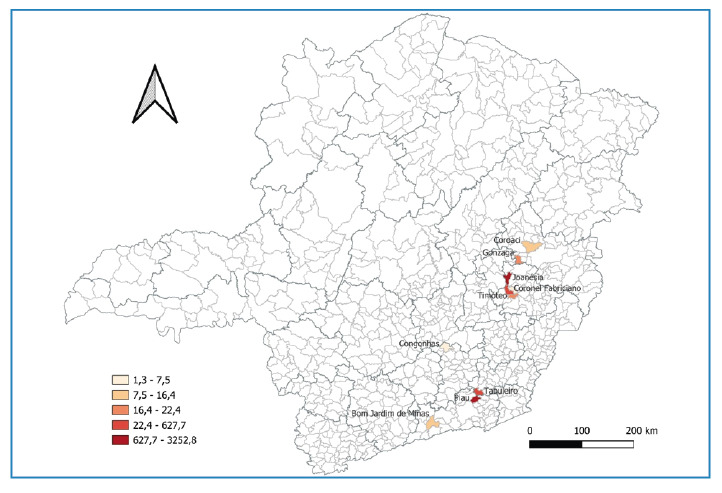
Spatial distribution of incidence per 100,000 inhabitants of confirmed Oropouche fever cases by municipality of residence, Minas Gerais, Brazil, 2024. **Notes:** Thicker lines indicate the boundaries of regional health units (RHU), whereas thinner lines represent municipal limits. This figure presents the data on cases reported in 2024, extracted from the official surveillance system on February 10, 2025.

The largest clusters of cases observed in the state were located in the municipalities of Joanésia (*n* = 140) and Piau (*n* = 84). The neighboring municipalities also reported confirmed cases, although in smaller numbers (Figure 3).

Ultimately, 144 individuals (51.8%) reported the presence of domestic or livestock animals in their households, workplaces, or study environments. Dogs were most frequently mentioned (126), followed by chickens (54), cattle (48), horses (38), cats (37), ducks (2), bees (1), goats (1), and pigs (1). In addition, 13 notifications (5.1%) included reports of non-accidental deaths of sylvatic or peridomestic animals (such as monkeys, birds, armadillos, anteaters, and sloths) near areas of residence or work.

An exploratory analysis was performed to compare the frequencies of key symptoms and selected clinical features between female and male patients. Fever was highly prevalent in both groups; however, the difference was not statistically significant. However, headaches (83.6% vs. 68.0%, *p* < 0.01), myalgia (76.3% vs. 64.0%, *p* < 0.05), and gastrointestinal symptoms (65.1% vs. 52.0%, *p* < 0.05) were significantly more frequent in females. Dysgeusia (52.0% vs. 32.8%, *p* < 0.01), pruritus (35.5% vs. 16.6%, *p* < 0.01), and skin lesions (28.3% vs. 12.0%, *p* < 0.01) were more common in females. The detailed results are provided in Supplementary Table S1.

**TABLE S1:** Clinical profile of confirmed Oropouche fever cases by sex, Minas Gerais, Brazil, 2024 (*N* = 277).

Signs and symptoms	**Female (*n* = 152)**	**Male (*n* = 125)**	X^2^	Prevalence ratio (95% CI)
Fever (%)	130 (85.5)	101 (80.8)	1.106	0.94 (0.85-1.05)
Headache (%)	127 (83.6)	85 (68.0)	9.238**	0.81 (0.71-0.94)
Myalgia (%)	116 (76.3)	80 (64.0)	5.028*	0.84 (0.72-0.98)
Gastrointestinal symptoms (%)	99 (65.1)	65 (52.0)	4.897*	0.80 (0.65-0.98)
Retro-orbital pain (%)	82 (53.9)	58 (46.4)	1.563	0.86 (0.68-1.09)
Ageusia (%)	80 (52.6)	57 (45.6)	1.357	0.87 (0.68-1.11)
Arthralgia (%)	71 (46.7)	57 (45.6)	0.034	0.98 (0.76-1.26)
Chills (%)	68 (44.7)	52 (41.6)	0.275	0.93 (0.71-1.22)
Dysgeusia (%)	79 (52.0)	41 (32.8)	10.270**	0.63 (0.47-0.85)
Dizziness (%)	53 (34.9)	39 (31.2)	0.416	0.89 (0.64-1.26)
Fatigue or weakness (%)	49 (32.2)	38 (30.4)	0.107	0.94 (0.66-1.34)
Malaise	43 (28.3)	36 (28.8)	0.009	1.02 (0.70-1.48)
Back pain	42 (27.6)	35 (28.0)	0.005	1.01 (0.69-1.48)
Pruritus	54 (35.5)	22 (16.6)	11.071**	0.50 (0.32-0.77)
Skin lesions	43 (28.3)	15 (12.0)	10.994**	0.42 (0.25-0.73)
Photophobia	22 (14.5)	8 (6.4)	4.630*	0.44 (0.20-0.96)
Flu-like or upper respiratory symptoms	18 (11.8)	10 (8.0)	1.114	0.68 (0.32-1.41)
Nuchal pain	16 (10.5)	12 (9.6)	0.065	0.91 (0.45-1.86)
Conjunctivitis	10 (6.6)	5 (4.0)	0.891	0.61 (0.21-1.73)
Dark urine	7 (4.6)	7 (5.6)	0.141	1.22 (0.44-3.37)
Shortness of breath	7 (4.6)	6 (4.8)	0.006	1.04 (0.36-3.02)

**Note:** This table presents data on cases reported in 2024 extracted from the official surveillance system on February 10, 2025. * *p*-value < 0.05, ** *p*-value < 0.01, *** *p*-value < 0.001

The same key symptoms considered in the analysis of female and male patients were considered in the evaluation of possible age-related differences. Only patients with retroorbital pain had a significantly lower median age (37 vs. 44 years, U = 10,971; *p* = 0.038). No statistically significant differences were reported in other signs and symptoms.

In addition, isolated reports of fainting, ageusia, burning sensation, confusion, facial edema, hematemesis, erythematous itchy lesions, rib pain, altered taste perception, drowsiness, swelling, paresthesia in the extremities, tremors, arrhythmia, leg fatigue, blurred vision, palpitations, dysuria, and listlessness were reported.

## DISCUSSION

The findings of this study highlighted the occurrence of OF outbreaks in Minas Gerais by 2024. This investigation fills an important gap in the epidemiological literature, as OROV infection has historically been restricted to the Amazon region, but has recently expanded to other areas of Brazil^7^ ^-^ ^9^, representing an emerging public health challenge^12^.

Several factors likely contribute to the geographic expansion of OROV beyond the Amazon basin, including the increasing distribution of vector species^1^ ^,^ ^4^ ^,^ ^7^, climatic and environmental changes^6^ ^-^ ^9^, human and animal migration^4^ ^,^ ^9^, underreporting and diagnostic confusion with other arboviruses^6^ ^,^ ^15^ ^-^ ^17^, urbanization, and socioeconomic vulnerabilities^7^ ^.^ ^9^. In 2024, autochthonous cases were reported in all 27 federative units of Brazil, spanning the northeast, southeast, central-west, and southern regions^12^, underscoring the nationwide spread of the virus.

In Minas Gerais, more cases have been reported among females than males, with a broad age distribution, predominantly among young adults. The literature does not indicate a consistent sex- or age-related pattern of infection, though Scachetti et al. (2024) found that individuals aged 20-59 years, regardless of sex, presented the highest incidence rates compared to the national cumulative incidence of 5.22 cases per 100,000 inhabitants.

Spatial analysis revealed a bimodal distribution of cases in the two municipalities, with Joanésia showing a peak around epidemiological week 15, and Piau around week 50 in 2024. The predominance of cases in rural and peri-urban areas suggests that transmission is associated with ecologically favorable environments for vector proliferation, despite the urbanized landscape of the state. *Culicoides paraensis*, the primary vector, thrives in humid habitats rich in decomposing organic matter such as banana or cacao debris^4^. Similar patterns have been described in Iquitos, Peru, where peri-urban transmission cycles have been identified, and most OROV isolates were obtained from rural and suburban areas^18^. 

The temporal distribution of cases observed in this study indicated possible seasonal transmission, consistent with increased vector reproduction in humid or flooded environments, coinciding with the rainy season in the Minas Gerais^6^ ^,^ ^12^ ^,^ ^19^ ^,^ ^20^. These findings highlight the need for continuous entomological surveillance and predictive models that integrate climatic and ecological data.

Clinically, fever, headache, myalgia, and retro-orbital pain were the most frequently reported symptoms, which is in agreement with the findings from other endemic areas. The occurrence of symptom recurrence in over one-quarter of the cases is noteworthy, suggesting that OROV infection may have a prolonged impact on patient well-being and productivity. Comorbidities such as hypertension and diabetes are common and may influence both the course and perception of illness.

The clinical presentation of OF is non-specific and overlaps with that of other arboviral infections, complicating the differential diagnosis. Studies have described a wide spectrum of symptoms, including fever, headache, myalgia, arthralgia, chills, dizziness, photophobia, nausea, vomiting, rash, and conjunctivitis^1^ ^,^ ^7^ ^,^ ^8^ ^,^ ^21^ ^,^ ^22^. Although typically self-limiting, OF can occasionally progress to neurological involvement such as meningitis or meningoencephalitis^2^ ^,^ ^3^ ^,^ ^12^ ^,^ ^23^. In the present study, no severe neurological manifestations were observed in confirmed cases.

With regard to pregnancy, the two confirmed cases evolved without complications. Although the vertical transmission of OROV is rare, it has been documented in the literature, including reports of fetal death and congenital anomalies^3^, underscoring the importance of monitoring this group in future investigations.

The high proportion of individuals who reported contact with domestic or livestock animals raises the hypothesis that environmental factors contribute to viral maintenance or amplification. Furthermore, reports of wildlife deaths, although limited, warrant further investigation into the possible role of sylvatic vertebrates as reservoirs or sentinels in OROV transmission cycles^4^ ^,^ ^5^.

This study had certain limitations that should be considered when interpreting the findings. The dependence on surveillance notifications and laboratory confirmations may underestimate the true incidence of OROV infection. In addition, spatial differences in testing availability and health infrastructure could have influenced case distribution. Nevertheless, this investigation has significant strengths, including the detailed clinical, epidemiological, and spatial characterization of OROV cases in non-endemic regions.

These results provide valuable insights into the ongoing expansion of Oropouche fever in Brazil and highlight the need for strengthened surveillance systems, differential diagnostic capacities, and integrated vector control strategies. Furthermore, the evidence generated contributes to the understanding of the social and environmental determinants of OROV transmission and supports the development of effective public health policies to mitigate its spread.
